# Clinical Profile, Severity Spectrum, and Hospital Outcome of Dengue Patients in a Tertiary Care Hospital in Dhaka City

**DOI:** 10.7759/cureus.28843

**Published:** 2022-09-06

**Authors:** Sadia Islam, Md Nazmul Hasan, Sumiya B Kalam, Md Shahnoor Islam, Md Jahid Hasan, Chowdhury Adnan Sami, Fazle R Chowdhury

**Affiliations:** 1 Medicine, Delta Medical College and Hospital, Dhaka, BGD; 2 Internal Medicine, Bangabandhu Sheikh Mujib Medical University, Dhaka, BGD; 3 Epidemiology and Public Health, Pi Research Consultancy Center, Dhaka, BGD

**Keywords:** dengue fever, severe dengue, outbreak in dhaka, mortality, dengue

## Abstract

Background

Bangladesh saw its most severe dengue outbreak in 2019, with the highest number of deaths reported. This study investigated the clinical characteristics, severity spectrum, and potential outcomes of dengue in patients admitted to a tertiary care institution in Dhaka.

Methods

This prospective observational study was done between May 2019 to April 2020. A total of 478 nonstructural protein 1 (NS1) antigen positive confirmed dengue cases were finally enrolled. The dengue patient's stratification and severity grading were performed according to the World Health Organization (WHO) dengue guidelines, for diagnosis, treatment, prevention, and control (2009). In addition, in-hospital outcomes were assessed in terms of mortality.

Results

The patient’s mean age was 33.90±15.82 (SD) years. The male-to-female ratio was 1.23:1. In addition to fever, the most common symptoms were myalgia (67.78%) and rashes (58.58%). According to WHO classification 33.90% of patients (n=162) were in group A, 49.40% (n=236) were in group B, and 16.70% (n=80) were in group C. The overall mortality was 1.23% in groups A, 2.97% in group B, and 11.25% in group C. The mortality was higher in the more severe group with statistical significance (p<0.001). The mean hospital stay time was significantly less in the surviving group (survival vs. death: 3.07±1.78 vs. 5.61±3.13 SD {days}, p<0.001). Leukopenia and thrombocytopenia were commonly seen in all of the severity groups.

Conclusion

Dengue epidemics are increasing in Bangladesh. Many group B and C cases are fatal. To reduce mortality and morbidity, health care providers must remain alert. This challenge requires public health interventions and hospital readiness.

## Introduction

Dengue has appeared as a significant public health issue in Asia since 1950. Among dengue's global "at risk" population, South East Asian countries contribute more than half of it [1]. The most vulnerable countries include Bangladesh, India, Pakistan, and Sri Lanka [1,2]. Before 2000, dengue viral illness occurred sporadically in Bangladesh, and an epidemic of 5,551 cases and 93 deaths were documented. However, since the first documented outbreak of dengue fever in Bangladesh in 2000, the number of hospitalized patients has increased to a point where it has exceeded 3,000 patients on six separate occasions; the highest was 10,148 in 2018 [3]. In 2019, the dengue case burden was the worst, with 10,1354 cases and 179 deaths spreading throughout the country and globally [4].

Dengue is an Aedes mosquito-borne viral disease brought about by an arbovirus [1]. The dengue virus (DENV) is classified into four serotypes named (dengue virus-1) DENV-1, (dengue virus-2) DENV-2, (dengue virus-3) DENV-3, and (dengue virus-4) DENV-4 [5]. However, the dengue serotypes share comparable antigenic characteristics, and cross-protection is only offered for a few months [6]. In Bangladesh, the prevalent serotypes of the dengue virus are DENV-2 and DENV-3, with the highest number of reported cases attributed to DENV-3 in most recent times [3]. In 2017, the re-emergence of DENV-3 was identified with a dramatic surge of dengue cases in the monsoons of 2018 and 2019 [7].

According to World Health Organization (WHO), the disease can be classified into group A, group B, and group C, where group C is the most severe form of the disease [8]. The mechanism that leads to the severe manifestation of DENV infections are multifocal, involving viral virulence factors and detrimental host response, resulting in abnormal hemostasis and increased vascular permeability. Secondary infection or multiple infections with different serotypes enhance the chances of more severe forms of diseases, such as dengue shock syndrome [6]. It was evident that the most frequent combination was DENV-2 and DENV-3, followed by DENV-1 and DENV-3. A combination of DENV-1, DENV-2, and DENV-3 was also identified. The higher prevalence of more severe cases may be due to secondary infection by serotype DENV-3 in 2018 and 2019 [7]. However, data related to severity are relatively less evident, and outcomes according to severity remain sparse in Bangladesh. Consequently, the current study investigated the severity and impact of dengue patients treated in a specialized hospital in Dhaka, Bangladesh, in 2019.

## Materials and methods

Study site, study population, and study participants

**Figure 1:**
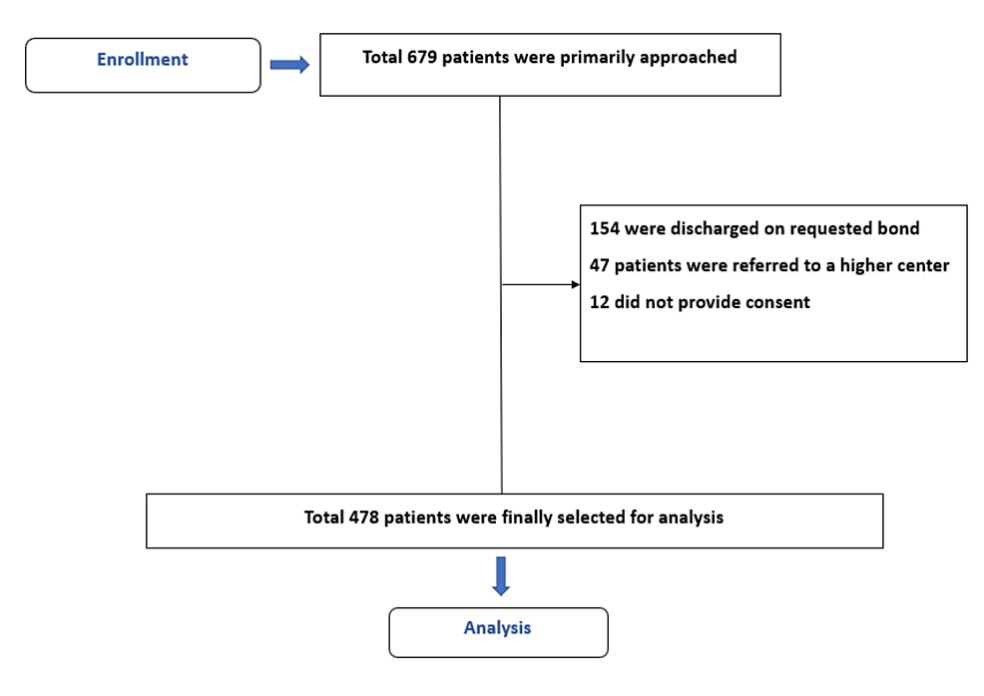
Flow chart of patient selection process.

The prospective observational study was conducted in the Department of Medicine, Delta Medical College and Hospital, Dhaka, one of the teaching hospitals situated in the capital, for 12 months from May 2019 to April 2020. A suspected case of dengue was defined by any person living or traveling in the dengue-endemic zone with fever (≥99°F) along with any two of the following symptoms: nausea or vomiting, rash, aches and pains, a positive tourniquet test, leukopenia (<4,000/mm^3^), and any warning signs. In addition, a confirmed dengue case was defined by a positive nonstructural protein 1 antigen (NS1Ag) in the blood of a suspected dengue patient. This approach was based on WHO classification for dengue (2009) group A (dengue without warning signs), group B (dengue with warning signs), and group C (severe dengue fever) [4]. A total of 679 patients were primarily approached, and finally, 478 patients were recruited as per inclusion and exclusion criteria. A detailed description of the patient's selection is described in (Figure 1).

Details of data collection

An adequate history, clinical examination, and investigations ruled out dengue co-infection with other viral, protozoal, or bacterial diseases. For data collection, a complete case record form was created and utilized throughout the data collection. The questionnaire included demographic information, clinical data, pertinent investigations, and the patient's admission outcome. Age, gender, first symptoms, underlying co-morbidities, clinical findings, laboratory investigations, and results were documented. Standard of care investigations, including total blood count, hematocrit, anti-dengue antibody IgM and IgG, alanine aminotransferase (ALT), aspartate aminotransferase (AST), and other laboratory assays, were conducted for all patients. Every patient underwent an immediate clinical examination focused on fever, vital signs, urine output, rash, and further necessary systemic examinations. Every patient was followed up every day till they were discharged. According to the WHO classifications of dengue (2009), disease severity was defined as group A, group B, and group C for all patients [4]. The patient's outcome was measured in terms of mortality during hospital admission. Data collection was performed by the lead researcher and was recorded in a Microsoft Excel sheet separately.

Laboratory investigations

Automated hematology analyzers, Siemens ADVIA® 2120 (Munich, Germany: Siemens), were calibrated every six months to standardize results when doing complete blood counts. For dengue NS1Ag detection, commercially available kits were utilized (InBios Dengue NS1 Detect kit; Seattle, Washington: InBios International, Inc.). According to the manufacturer, sensitivity and specificity of the kit are 86.6% positive percent agreement (PPA) and 97.8% negative percent agreement, respectively, with prospectively collected positive and negative confirmed clinical specimens.

Statistical analysis

Statistical studies were carried out utilizing SPSS version 20.0 for Windows (Armonk, NY: IBM Corp.). We used both descriptive and inferential statistics. Categorical variables were expressed as frequencies and percentages, whereas continuous variables were expressed as the mean and standard deviation. In addition, one-way ANOVA was performed on the continuous variables, and the chi-square test was used for the categorical variables. The result was calculated using a 95% confidence interval, and p<0.05 was deemed statistically significant.

Ethical consideration

Before beginning the trial, the Delta Medical College and Hospital Ethical Review Committee (ERC) examined and approved the study protocol. Before participation, informed consent was obtained from the patients or their legal guardians if the patients were minors. Ethical measures were maintained throughout the study period, and a high standard of confidentiality and anonymity of the participants were preserved. All of the study procedures complied with the latest Declaration of Helsinki.

## Results

**Figure 2:**
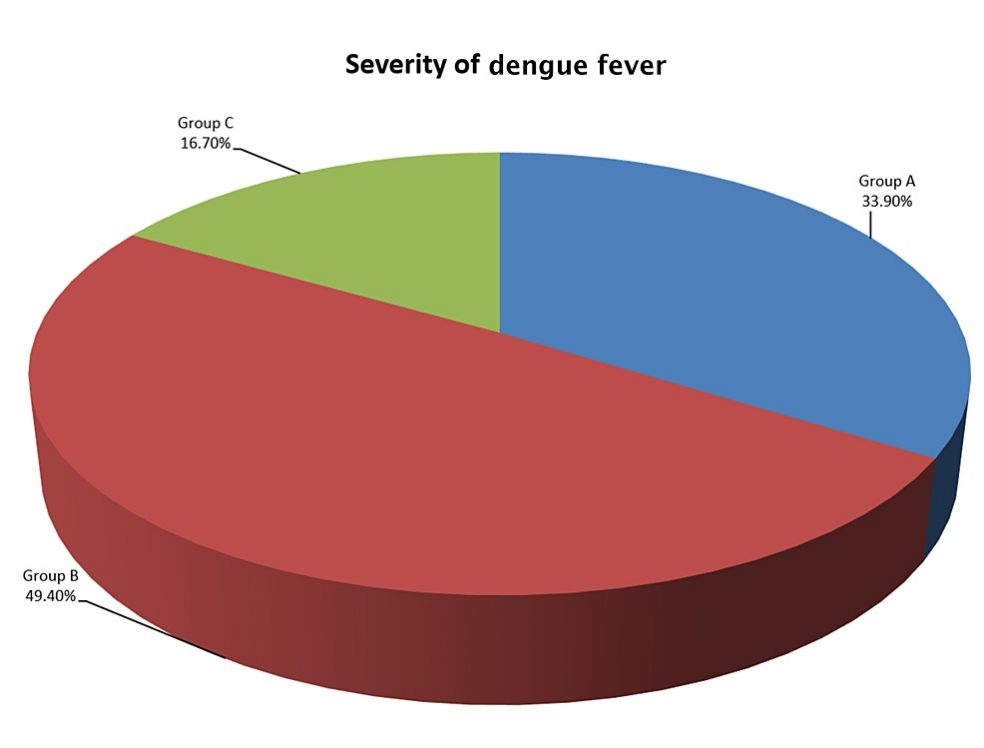
Severity of dengue patients (n=478).

The average age of the study participants was 33.90±15.82 (mean±SD) years. The number of male patients was slightly higher than that of female patients (the overall male-female ratio was 1.23:1). Dengue patients with severe grading (grade C) are relatively older than other groups (p<0.001), and severity increases with age (p<0.001). Most patients in each group were aged 18-30 years (Table 1). Total 478 confirmed dengue cases were distributed in following way: group A - n=162, 33.90%; group B - n=236, 49.40%; and group C - n=80, 16.70% (Figure 2).

**Table 1:** Demographic profile of patients (n=478). *The mean age of group C patients was significant in comparison to group A. **The mean age of group C patients was significant in comparison to group B. The p-value was determined by one-way ANOVA and chi-square test. Post hoc analysis with the Bonferroni method was performed.

Variable		Total	Severity of dengue fever			p-Value
		(n=478)	Group A (n=162)	Group B (n=236)	Group C (n=80)	
Age	Mean±SD (years)	33.90±15.82	30.59±13.13	34.08±15.99	40.09±18.36*,**	<0.001
Age group (years) (n, %)	<18	37 (07.70)	18 (11.10)	16 (06.80)	03 (03.80)	0.002
	18-30	231 (48.30)	82 (50.60)	121 (51.30)	28 (35.00)	
	31-40	86 (18.00)	34 (21.00)	35 (14.80)	17 (21.30)	
	41-50	50 (10.50)	15 (09.30)	27 (11.40)	08 (10.00)	
	51-60	34 (07.10)	06 (03.70)	17 (07.20)	11 (13.80)	
	>60	40 (08.40)	07 (04.30)	20 (08.50)	13 (16.30)	
Gender (n, %)	Male	264 (55.20)	88 (54.30)	130 (55.10)	46 (57.50)	0.895
	Female	214 (44.80)	74 (45.70)	106 (44.90)	34 (42.50)	

Fever was the most common symptom (94.56%), followed by myalgia (67.78%), rashes (58.58%), bone pain (54.39%), headache (48.95%), petechiae (42.89%), vomiting (32.01%), abdominal pain (29.71%), and cough (24.90%). Gastrointestinal (GIT) bleeding (13.18%), diarrhea (11.92%), gum bleeding (10.25%), hematuria (09.41%), and retro-orbital pain (08.58%) were evident. The prevalence of bone pain, myalgia, cough, headache, vomiting, diarrhea, drowsiness, gum bleeding, petechiae, GIT bleeding, and hematuria was significantly (p<0.05) higher in groups C than in groups A and B. In contrast, abdominal pain was significantly higher in group B than in groups A and C (Table 2).

**Table 2:** Clinical manifestation of dengue cases.

Clinical features (n, %)	Total (n=478)	Severity grading of dengue fever			p-Value
		Group A (n=162)	Group B (n=236)	Group C (n=80)	
Fever	452 (94.56)	151 (93.21)	221 (93.64)	80 (100)	0.062
Abdominal pain	142 (29.71)	00 (00)	112 (47.50)	30 (37.50)	<0.001
Retro-orbital pain	41 (08.58)	16 (9.90)	19 (08.10)	06 (07.50)	0.759
Bone pain	260 (54.39)	47 (41.40)	140 (59.30)	53 (66.30)	<0.001
Myalgia	324 (67.78)	96 (59.30)	167 (70.80)	61 (76.30)	0.011
Cough	119 (24.90)	23 (14.20)	68 (28.80)	28 (35.00)	<0.001
Headache	234 (48.95)	71 (43.80)	110 (46.60)	53 (66.30)	0.003
Rashes	280 (58.58)	87 (53.70)	149 (63.10)	44 (55)	0.133
Vomiting	153 (32.01)	21 (13)	87 (36.90)	45 (56.30)	<0.001
Diarrhea	57 (11.92)	11 (6.80)	32 (13.60)	14 (17.50)	0.030
Drowsiness	09 (01.89)	00 (00)	00 (00)	09 (11.30)	<0.001
Gum bleeding	49 (10.25)	00 (00)	33 (14.00)	12 (15)	<0.001
Petechiae	205 (42.89)	55 (34.00)	103 (43.60)	47 (58.80)	0.001
GIT bleeding	63 (13.18)	00 (00)	39 (16.50)	30 (37.50)	<0.001
Hematuria	45 (09.41)	00 (00)	30 (12.70)	15 (18.80)	<0.001

Although the proportion of patients with leucopenia was comparable between groups, the number of patients with leukocytosis and platelet count <50,000/mm^3^ was considerably greater in group C than in groups B and A (Table 3). The overall mortality rate of dengue fever was 3.77%. The proportion of mortality and severity of dengue fever was statistically significant (Table 4).

**Table 3:** Baseline leucocyte and platelet counts according to severity grading.

Lab findings	Total (n=478)	Severity grading of dengue			p-Value
		Group A (n=162)	Group B (n=236)	Group C (n=80)	
Leukopenia (<4,000/mm^3^)	250 (52.30)	79 (48.80)	126 (53.40)	45 (56.30)	0.491
Leukocytosis (>11,000//mm^3^)	10 (02.09)	00 (00)	04 (01.70)	06 (07.50)	0.001
Platelet count (<50,000/mm^3^)	277 (57.95)	48 (29.60)	149 (63.10)	80 (100)	<0.001

**Table 4:** Outcome of the patients with severity grading.

Severity of dengue fever	Outcome		p-Value
	Death (n, %)	Survive (n, %)	
Group A (n=162)	02 (01.23)	160 (98.77)	<0.001
Group B (n=236)	07 (02.97)	229 (97.03)	
Group C (n=80)	09 (11.25)	71 (88.75)	
Total (n=478)	18 (03.77)	460 (96.23)	

The average length of hospital stays for patients who died was 5.61±3.13 days, while the average duration for those who survived was 3.07±1.78 days. Those who survived had a shorter hospital stay than patients who died (p<0.001) (Table 5).

**Table 5:** Outcome of the patients with duration of hospital stay. *P-value is determined by unpaired t-test. **P-value is determined by chi-square test.

Variable		Outcome		p-Value
		Death	Survive	
Duration of hospital stay, (mean±SD) (days)		5.61±3.13	3.07±1.78	<0.001*
Duration (n, %)	≤5 days	10 (55.56)	411 (89.34)	<0.001**
	>5 days	08 (44.44)	49 (10.65)	

## Discussion

Dengue fever, caused by the dengue virus, was relatively uncommon in Bangladesh before the first outbreak occurred in 2000, but it is now quite common. In addition, it is known for its increased morbidity and mortality. Therefore, our study aimed to determine the severity spectrum and outcome of dengue patients in a private hospital in Dhaka.

Among the 478 study participants, 49.40%, 33.90%, and 16.70% had group B, A, and C dengue fever, respectively. Flamand et al. found a high proportion of group B cases comprising 68% of their participants [9]. Group C patients (40.09±18.36 years) were significantly older than others. The overall mean age (33.90±15.82 years) is consistent with the findings of Ahmed et al. (32.22 years) and Laul et al. (31.36±13.17 years) [10,11]. Consistent with the results of Kumar et al. and Azad et al., this study also noted a male majority (48.30%) with a male-to-female ratio of 1.23:1 [12,13]. However, Farhana et al. found a reverse ratio of genders (male-to-female ratio of 0.84:1) [14].

Several factors have been identified for the increased prevalence of dengue viral illness in urban and semi-urban areas. Construction, lifestyle changes, poor water management, inefficient water storage, and stagnant rainwater in outdoor containers lead to vector breeding grounds [15]. In recent years, Dhaka became a city of mass developmental activities that might contribute to the enhancement of the breeding place of Aedes mosquitoes, thus probably facilitating the re-emergence of dengue viral illness.

The illness is characterized by fever, arthralgia, aches and pains, rash, subconjunctival hemorrhages, respiratory symptoms, gastrointestinal disorders, and other symptoms. Asymptomatic infection also contributes significantly to the total cases [10,16]. Several atypical characteristics were also seen in this outbreak, such as pleural effusion, neurological manifestations, encephalopathy even Guillain-Barré syndrome (GBS) and myocarditis are also reported which were included in the expanded dengue syndrome category [10,15]. These, along with the usual complications of dengue, contribute to increased case fatality and prolonged hospital stays, ultimately a higher number of intensive care procedures. Additionally, overcrowding of tertiary care centers or admission to specialized centers for more intensive management was also noticed in this outbreak [17].

Concordant with the findings of other similar studies, this study also revealed fever, myalgia, rashes, bony pain, and headache as the most frequent clinical presentations of dengue [10,18]. Different gastrointestinal tract (GIT) symptoms, such as vomiting and abdominal pain, were observed in 32.01% and 29.71% of cases, possibly due to dengue virus-mediated liver injury [12,16]. Bleeding manifestations such as petechiae, GIT bleeding, gum bleeding, and hematuria were observed in 42.89%, 13.18%, 10.25%, and 09.41% of cases, respectively, which could be due to the consequences of thrombocytopenia and imbalance between clotting and the fibrinolysis system [19-21].

Our study found a platelet count of <50,000/mm^3^ in 57.95% of cases, leukopenia in 52.30% of cases, and leukocytosis in 2.09% of cases. Bone marrow suppression, ineffective thrombopoiesis, and immune-mediated peripheral destruction of platelets are some of the reasons for thrombocytopenia and leukopenia in dengue patients [22-24]. In addition, the prevalence of bone pain, myalgia, cough, headache, vomiting, diarrhea, drowsiness, gum bleeding, petechiae, GIT bleeding, and hematuria was significantly (p<0.05) higher in group C than in groups A and B. Similarly, the number of patients with leucocytosis and low platelet count (<50,000/mm^3^) was significantly higher in group C than in group B. All these findings are nearly concordant with the results of Tsai et al. [25].

The mortality rate of dengue fever was 3.77%. The rate is relatively higher than national estimates in 2019 (1.7%) [4]. This high death rate might be explained by the hospitalization of relatively severe cases, among whom this study was conducted. In addition, other studies reported similar death rates to ours (3.9%) [11]. This study also noted the highest mortality in group C (11.25%), followed by group B (02.97%) and group A (01.23%). Shock leading to multiorgan failure, acute respiratory distress, hepatic encephalopathy, and encephalitis are reasons for the higher mortality rate in group C than in groups A and B. In the severe dengue group, the existence of plasma leakage, fluid buildup, respiratory difficulty, severe bleeding, or involvement of multiple organs might explain this finding [26].

This study has significant limitations, including missing data on liver enzymes due to the higher center referral and early discharge on request. There is no available data on serotyping of the virus in the studied patient. In addition, the overall number of children was small, likely because many of the children's consultations might have been diverted to specialized hospitals for children in the city. Also, we could not perform a survival analysis due to the lack of follow-up data.

## Conclusions

Dengue seems to be a continuous threat in Bangladesh with increasing epidemicity. A good number of cases are presented as groups B and C, with a high chance of fatality. Health care providers need to be more vigilant while encountering a case so that mortality and morbidity can be minimized. Appropriate public health measures and hospital preparedness are vital to facing this challenge.
